# Closing Editorial: Peer teaching and learning; the key to scholarship

**DOI:** 10.15694/mep.2017.000169

**Published:** 2017-09-28

**Authors:** Jamie Read, Julie Browne

**Affiliations:** 1 CAMERA; 2 Centre for Medical Education

**Keywords:** peer learning

## Abstract

This article was migrated. The article was marked as recommended.

In our opening editorial we discussed our desire to receive a diverse range of submissions regarding peer teaching and learning. We are delighed to have received such a range of different submissions from healthcare education teams across the globe, focussing on peer education in very different settings. In this, our closing editorial, we discuss the key findings from the submissions that we have received, provide our own reflections on what authors have submitted and discuss our experiences of co-editing this themed edition of MedEdPublish.

## Introduction

Following a really excellent AMEE 2017 meeting in Helsinki in August this year, we were both struck once again by the extraordinarily powerful benefits offered by peer learning and teaching. Everywhere we went within the conference, excited and energetic conversations were being exchanged; ideas and information were flowing back and forth; constructive feedback offered and received; new networks and social connections created; and original and innovative ideas exchanged. This is peer teaching and learning at its very best.

Peer teaching and learning is an essential part of creating and maintaining a scholarly educational community for the benefit of everyone, and in particular the patients whom we ultimately serve. But, as we outlined in our opening editorial (1) on this theme, although effective peer-to-peer social learning in action is going on all around us (and a conference like AMEE is just one manifestation of this) it’s incredibly difficult to capture its wonderful complexity and vibrancy, or to really get to grips with its amazing potential for transformative learning.

Despite this, we think that MedEdPublish, and this theme issue in particular, have gone a long way towards doing just that. We have published sixteen papers celebrating the diversity and energy of the field; and if most of these have not yet been able to demonstrate or quantify conclusively the learning gains that peer teaching and learning offer (especially as these are manifested in effective clinical care), they all have at least added to our store of knowledge around what is being done in the field, by whom, and where, and given us a flavour of the great work going on in clinical and higher education settings worldwide.

## Articles submitted to this themed issue

It has been a real pleasure to read so many articles about the different approaches to near-peer education that are being employed. In particular, it has been exciting to read submissions to the journal theme from so many different countries and different types of healthcare setting.

We would like to thank those who have submitted articles to this themed issue of MedEdPublish for the time that they have taken in writing up their research, their generous and thoughtful reflections on what they have achieved, and the care they have taken to report honestly the feedback they have received from learners who have been involved in the process. From detailed curricula evaluations (2) to reflections from staff (3,4) and students (3-5) involved in teaching it has been heartening to read about the positive impact that near-peer education has had on the learning process. In particular, it has been exciting to read about the variety of approaches taken in so many different countries and different types of educational and healthcare settings, including medical, nursing and veterinary studies (5-7) basic science lectures,(8,9) work inductions (10), community settings (2) and even during intercalated degrees (3,11).

The breadth of the topics that have been submitted has also been of great interest. These topics have included teaching approaches with varying levels of interaction, different scientific and clinical areas and varying uses of technology (6). Ultimately we have seen that both faculty and learners appreciate the role of near-peer education in most topics and most areas, using a variety of approaches.

Near-peer education also seems to benefit those who teach too. We have seen from the reflective pieces that have been submitted that faculty find the process rewarding and that it helps to improve their own skills and knowledge (3,4), perhaps in a way that is not associated with a more traditional teaching style, where there is a greater ‘gap’ between teacher and learner.

Perhaps unsurprisingly, many of the papers submitted have involved near-peer education driven by transitions (7) or assessment, often high stakes examinations that students and junior doctors have designed near-peer educational interventions around to allow people to feel more prepared (4,12,13). The adage of ‘assessment drives learning’ might well be supplemented more contemporaneously by the statement that assessment drives near-peer education. Indeed, many of the most innovative approaches to such education were around medical school and postgraduate examinations.

It is clear that near-peer education offers significant benefits for both teachers and learners and that adequate institutional support greatly benefits and approach which is held in high regard from those who are involved.

## Our own reflections on the MedEdPublish process

*MedEdPublish* is very like a microcosm of the AMEE conference. It relies on goodwill and community to work effectively. If it weren’t for (a) those who put the effort into writing up and sharing their work with their peers, and (b) those who give friendly advice and feedback, very little learning or improvement in medical education practice would take place.

We would therefore like to thank the peer reviewers who have generously given up their time to provide perceptive, constructive and realistic feedback on the papers we have selected for publication. They are particularly important to a journal such as *MedEdPublish*, especially in view of the reluctance of more traditional journals to publish small scale studies, pilots and local improvement projects.

Our own reflections on the process have been that:

• A post-publication peer-review process encourages a wider breadth of experiences to be shared, with the ability to share best practice in a way that more traditional journals do not always allow.
• The process encourages more junior educators to share their experiences and develop their careers with useful responses from other medical educators.
• As educators we should encourage such an approach, but it does require robust safeguards to be in place.
• In particular, the process is a risk of a lack of robustness and is reliant on rapid, high quality peer-review from experts in their field. Like all workplace-based assessment (and peer review is the ultimate academic WBA) it is important to make sure that it is much more than a tick-box exercise and improves learning for everyone involved (14).

So to summarise, do get involved, share your expertise and allow this innovative model to thrive with high quality peer review.

## Notes On Contributors

Julie Browne is a Senior Lecturer in Academic Practice at Cardiff University School of Medicine and course lead for the intercalated BSc in Medical Education. She is vice Chair of the Academy of Medical Educators Council and serves on a number of its standing committees including Education and Professional Standards. She is also a Senior Fellow of the Higher Education Academy. Her professional background is in academic publishing and she was formerly Managing Editor of *Medical Education* and *The Clinical Teacher.* In 2007 she co-ordinated the work of the Expert Advisory Panel on assessment for the Tooke Inquiry into Modernising Medical Careers. She is a GMC Education Associate, and is the author or co-author of a number of peer reviewed publications on medical education, including several chapters and a book on medical curriculum development. Her interests include educator development, medical humanities, publication ethics and curriculum theory. In 2015 she was awarded the President’s Silver Medal of the Academy of Medical Educators for outstanding and sustained contributions to medical education.

Dr Jamie Read is currently a PhD student with the CAMERA team at Plymouth University. Until recently he was an NIHR funded Academic Clinical Fellow with research interests in remediation in medical students and doctors and the role that near-peer education can play in both formal and informal training of medical students. His clinical background is as a trainee in Geriatric Medicine based in the South West of the UK, most recently at Derriford Hospital in Plymouth. Jamie has been involved in supporting early careers medical educators for some time and until recently he chaired the Early Careers Group of the Academy of Medical Educators - the professional organisation for educators of doctors, dentists and vets within the UK which has produced Professional Standards to support medical educators. He is now the Academy Registrar and Council member and retains a strong interest in encouraging more junior medical educators to develop their careers.

## Declarations

Julie Browne and James Read are guest Theme Editors for the AMEE MedEdPublish themed issue - Medical students and postgraduate trainees as medical educators.
