# Supplementary material for: Closing Editorial: Peer teaching and learning; the key to scholarship
Source: MedEdPublish (2016). 2017 Sep 28;6:169. [Version 1] doi: 10.15694/mep.2017.000169 (PMC10885299; doi:10.15694/mep.2017.000169)
Supplement: Peer review history

# Reviewer report: Reviewer response for version 1

**DOI:** 10.21956/mep.19353.r28292

**Published:** 2019-02-22

**Authors:** Trevor Gibbs

**Affiliations:** 1 AMEE

This review has been migrated. The reviewer awarded 5 stars out of 5 As curricula content becomes more and more important and teaching resource becomes increasingly scarce, this topic is very important. This closing editorial not only sums up how important the subject is to working educationalists but how diverse the various approaches are.What I felt was very encouraging from a students perspective was the confidence that peer-teaching gave, a confidence so important within today's health professions environment.Well done to the theme Editors for bringing together this excellent series of papers, thank you.

Reviewer Expertise:

NA

No decision status is available

# Reviewer report: Reviewer response for version 1

**DOI:** 10.21956/mep.19353.r28291

**Published:** 2017-11-01

**Authors:** Richard Hays

**Affiliations:** 1 James Cook University

This review has been migrated. The reviewer awarded 5 stars out of 5 I would like to thank the authors for their summary of the themed papers published during the July to September period. The submissions were diverse, well grounded and interesting - just what was desired. The comment that near-peer learning is heightened by looming assessments and transitions is both interesting and important. It fits with experience, because learners focus much of their effort on getting through 'barriers' and then being 'ready' for the next stage. These two concepts overlap substantially, particularly in the minds of educators, but may be regarded by learners as more separate, particularly if their assessments do not seem relevant to the desired learning outcomes. Is this an alignment issue? Further research into this topic may be worthwhile. I would also like to thank the authors for their reflections on the MedEdPublish submission and publication experience. Their insights are a valuable contribution to improving the journal's relevance to medical educators. We will strive to increase the interactive component of the website, encouraging more to contribute their thoughts on published papers.

Reviewer Expertise:

NA

No decision status is available
